# A Computational Study on Renal Artery Anatomy in Patients Treated with Fenestrated or Branched Endovascular Aneurysm Repair

**DOI:** 10.3390/bioengineering12050482

**Published:** 2025-05-01

**Authors:** Yuzhu Wang, Yuna Sang, Wendong Li, Minjie Zhou, Yushun Zhao, Xiaodong He, Chao Wang, Xiaoqiang Li, Zhao Liu

**Affiliations:** 1 Department of Vascular Surgery, Nanjing Drum Tower Hospital, Affiliated Hospital of Medical School, Nanjing University, Nanjing 210008, China; 602023350079@smail.nju.edu.cn (Y.W.); vasculars@163.com (W.L.); 2 School of Astronautics, Harbin Institute of Technology, Harbin 150080, China; ssangyuna@163.com (Y.S.); yushunzhao@hit.edu.cn (Y.Z.); chaowang@hit.edu.cn (C.W.); 3 Research and Development Department, Nanjing Cloudure Medical Co., Ltd., Nanjing 211200, China; eye333333@163.com; 4 National Key Laboratory of Science and Technology on Advanced Composites in Special Environments, Harbin Institute of Technology, Harbin 150080, China; hexd@hit.edu.cn

**Keywords:** F/B EVAR, hemodynamics, prosthetic vascular stent, branch flow

## Abstract

(1) Background: Renal artery occlusion after F/B EVAR for abdominal aortic aneurysm is a serious complication that may require re-intervention, and understanding the hemodynamic mechanisms by which it occurs is essential to optimize the surgical procedure. (2) Methods: We used computational fluid dynamics (CFD) to analyze the impact of various parameters on blood flow. Theoretical vascular models were constructed based on the common dimensions and angles of aortic stents and branch arteries in clinical practice. Actual case models were constructed from CT image data of six patients treated with F/B-EVAR. Data were collected for analysis after simulation and calculation by FLUENT software. (3) Results: Theoretical model simulations showed that a larger tilt angle of the branch stent, smaller branch entry depth, and larger branch stent diameter were beneficial for blood flow. In the case models, a significant difference in the tilt angle of the renal artery stents was observed between the high- and low-flow groups, while the differences in entry depth and branch stent diameter were not significant. Occluded renal arteries had lower WSS values than patent ones. (4) Conclusions: This study offers valuable guidance for optimizing stent placement in F/B EVAR to mitigate renal artery occlusion risk.

## 1. Introduction

Since endovascular aneurysm repair was applied to abdominal aortic aneurysms [1], this low-risk technique has become the way forward in the treatment of aortic disease. With the advent of the fenestrated or branched EVAR (F/B EVAR) technique [2], complex aortic diseases involving important branches can also be endovascular aneurysm repaired using this technique. This approach, although more difficult to perform, has the advantages of high branch arterial stent patency, less surgical trauma, fewer craniocerebral complications, and a low rate of endoleak.

One of the serious complications after F/B EVAR is renal artery occlusion, which can lead to reintervention. During surgery, branch artery stents inevitably enter a portion of the aortic stent, causing some impact on blood flow. The patency of branch arteries may be affected by inappropriate angles and entry lengths, although the exact conditions are unknown. This paper employs a computational fluid dynamics approach to analyze various scenarios of branch stent entry into the aorta in F/B EVAR, and the conclusions drawn are validated by actual cases.

## 2. Materials and Methods

### 2.1. Scene Setting and Model Building

Application scenarios are defined based on the common sizes and angles of aortic stents and branch arteries when performing F/B EVAR in clinical work. Modeling software was utilized to create a vascular model, and a 3D model of the aortic vessel with an artificial stent was constructed through stretching, resection, and release operations. To realistically simulate the blood flow in the aortic vessel after the placement of the artificial stent, this paper employed the widely used FLUENT finite element analysis software to conduct simulations and imported the fluid and solid domains into the software for hydrodynamic analysis.

**Figure 1:**
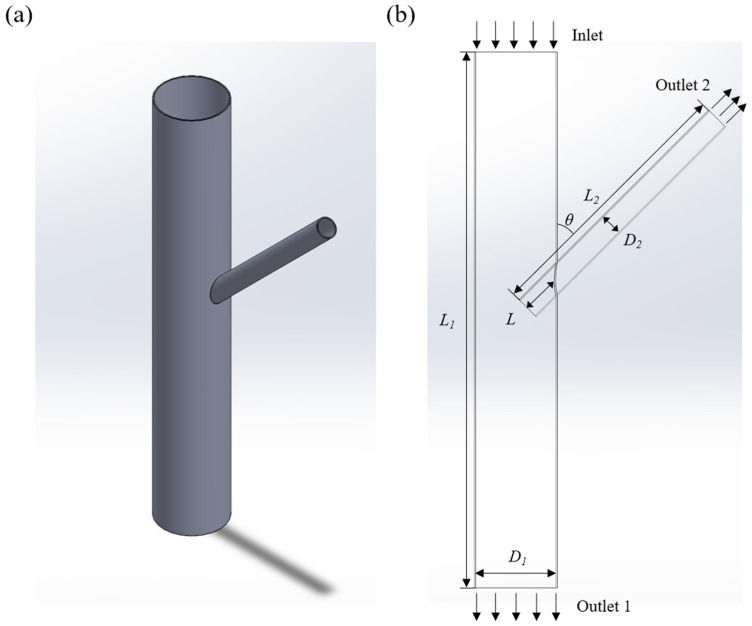
Vascular model: (**a**) geometrical model; (**b**) geometrical dimension.

To ensure the scientific accuracy of the calculation results, this paper optimized the aortic and branch artery stent models, as illustrated in Figure 1a. In the figure, D1 represents the aortic diameter, D2 is the diameter of the branch stent, L1 is the length of the aorta, L2 is the length of the branch stent, θ is the tilt angle of the branch stent, L represents the depth of branch entry, which indicates the extent to which the branch stent axis branches into the aortic vessel, and Vinlet represents the velocity of aortic inlet flow, as depicted in Figure 1b. Additionally, to ensure sufficient blood flow in both the aorta and branch arteries, this paper set the lengths of the aortic and branch stents at ten times their respective diameters, i.e., L1 = 200 mm and L2 = 100 mm.

### 2.2. Clinical Case Selection

Six patients diagnosed with abdominal aortic aneurysm at Drum Tower Hospital during 2018–2022 and treated with F/B-EVAR were recruited. Three of them developed renal artery occlusion during postoperative follow-up, and the other three did not. All six patients were male and were carefully selected to ensure that their postoperative levels of red blood cells, white blood cells, platelets, triglycerides, and cholesterol were within normal ranges, aiming for comparable blood viscosity. All six patients had primary suprarenal abdominal aortic aneurysms, with no history of open surgery or aortic surgery, and were indicated for surgery due to an aneurysm with a maximum diameter greater than 5.5 cm. The aortic stents were from Lifetech (Shenzhen, China), and the branch arterial stents were from Viabahn (Gore Medical, Flagstaff, AZ, USA).

### 2.3. Case Modeling and Analysis

**Figure 2:**
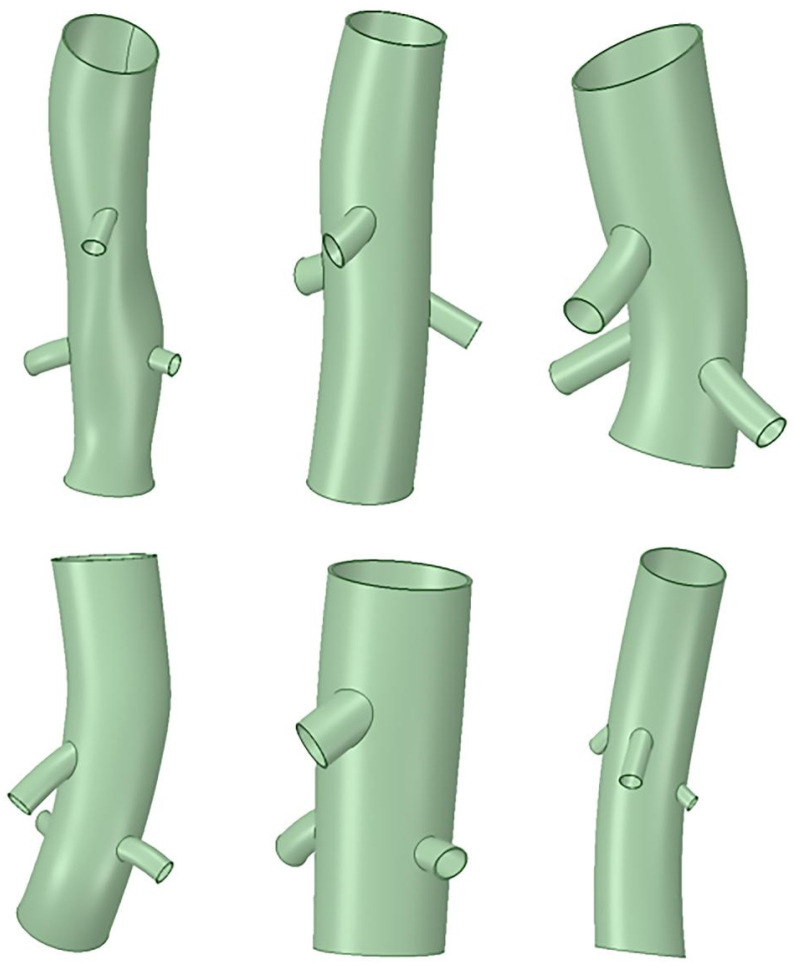
Three-dimensional modeling of six patient stents.

CT image data of the six selected patients after F/B EVAR of the abdominal aorta were imported into the Mimics medical image control system (Mimics^®^ Innovation Suite, Materialise NV, Leuven, Belgium). Binary masks of the abdominal aortic stent and branch arterial stent geometries were isolated and processed to obtain the corresponding surface mesh data. The surface mesh data were smoothed, and excess structures were removed using Geomagic Wrap 2014 (Rock Hill, SC, USA). A 3D model of the stent system for hemodynamic calculations was constructed using Geomagic Design Direct to morphologically fit the processed mesh data, as shown in Figure 2, and the final simulation was carried out by FLUENT (Ansys, Inc, Canonsburg, PA, USA) finite element analysis software, employing the same methodology as described above.

### 2.4. Boundary Condition Setting

In this paper, the blood flow is modeled as an incompressible Newtonian fluid with a density of 1055 kg/m^3^ and a viscosity coefficient of 0.0035 kg/(m·s). The blood flow basin boundary was set as a rigid wall without slip. The boundary condition at the entrance of the aortic vessel was defined by the inlet velocity, while at the exit of the aortic vessel and branch artery, it was set by the outlet pressure. The blood flow velocity in the middle segment of the human renal artery is known to range from 0.8 m/s to 1.2 m/s, and the blood pressure typically falls within the range of 73 mmHg to 100 mmHg. Since the blood flow pattern analyzed in this paper assumes constant flow, the inlet velocity and outlet pressure were both set to 0.8 m/s and 13,300 Pa, respectively.

### 2.5. Numerical Calculation and Data Analysis

CFD-POST (Ansys, Inc, Canonsburg, PA, USA) software was utilized to postprocess the results, employing the SIMPLE algorithm for the solver set to steady state. The parameters, such as flow rate, wall shear stress (WSS), and mass flow rate, were obtained under the specified boundary conditions. Calculations from actual case models were grouped according to renal artery mass flow, and normality was assessed with the Kolmogorov–Smirnov test, using the Student *t*-test for normally distributed continuous variables and the Mann–Whitney U test for non-normally distributed continuous variables. *p* values < 0.05 were considered significant for all calculations. Survival analysis was performed using Kaplan–Meier curves. Statistical analysis was performed using SPSS version 26 (IBM Statistics, Chicago, IL, USA).

## 3. Results

### 3.1. Theoretical Model Simulation Results

#### 3.1.1. Without the Branch Stent

This paper first analyzed the relationship between blood flow in the absence of the branch stent and the angle of inclination of the branch artery. The patency of the branch artery was assessed by measuring the mass flow rate at the branch exit cross-section; a positive flow rate indicates blood flowing out of the branch stent, while a negative rate indicates reflux. The presence of reflux suggests that the branching conditions at this point were highly unfavorable for normal blood flow, but this is a purely computational result obtained under ideal modeling conditions. Clinically, these branch arterial flows typically do not exhibit reflux but are more prone to slow flow, low flow, and even branch arterial occlusion under suboptimal branch access conditions.

**Figure 3:**
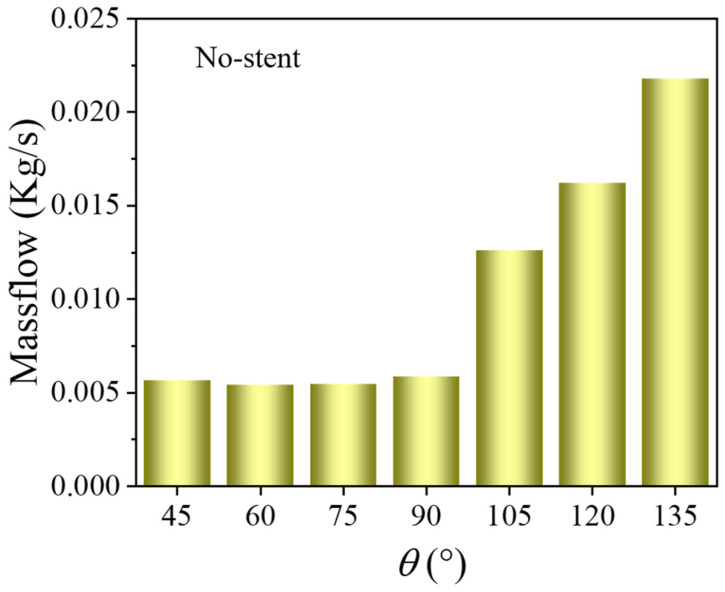
Branch flow between tilt angles without branch stents (D1 = 30 mm, D2 = 8 mm, Vinlet = 0.8 m/s).

Figure 3 shows the branch flow rate between the tilt angle without branch stents. Without branching stents, the blood flow in the branch stent is directed outward. When the angle is small (θ ≤ 90°), the outflow remains constant. However, when θ > 90°, the outflow increases with an increasing tilt angle.

**Figure 4:**
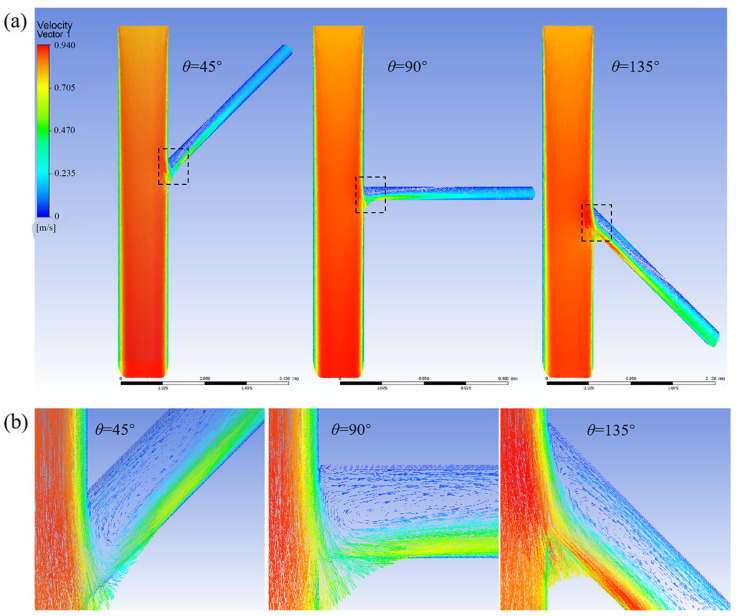
(**a**) Velocity distribution cloud for the model without branch stents; (**b**) Enlarged views of the branching area within the dashed box.

Figure 4 shows the flow velocity distributions for tilt angles of 45°, 90°, and 135°. A vortex phenomenon was observed at the upper end of the branch arteries and the vortex region decreased with an increase in the tilt angle.

#### 3.1.2. Effect of Different Tilt Angles and Branch Entry Depths on Blood Flow in Branch Arteries

**Figure 5:**
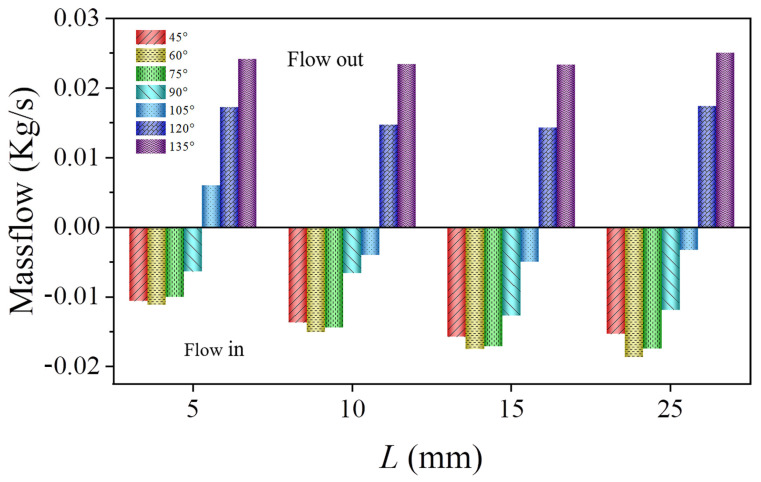
Branch flow as a function of tilt angle and branch entry depth (D1 = 30 mm, D2 = 8 mm, Vinlet = 0.8 m/s).

Figure 5 demonstrates how branch blood flow was affected by the tilt angle and depth of the branch stent. When the tilt angle of the branch stent was relatively small (θ ≤ 90°), blood flow in the branch stent exhibited reflux, and the reflux rate increased with an increase in the branch entry depth. For larger tilt angles (θ > 100°), the branch stent blood flow consistently remained in outflow, and the volume of outflow increased with a rise in the branch entry depth.

**Figure 6:**
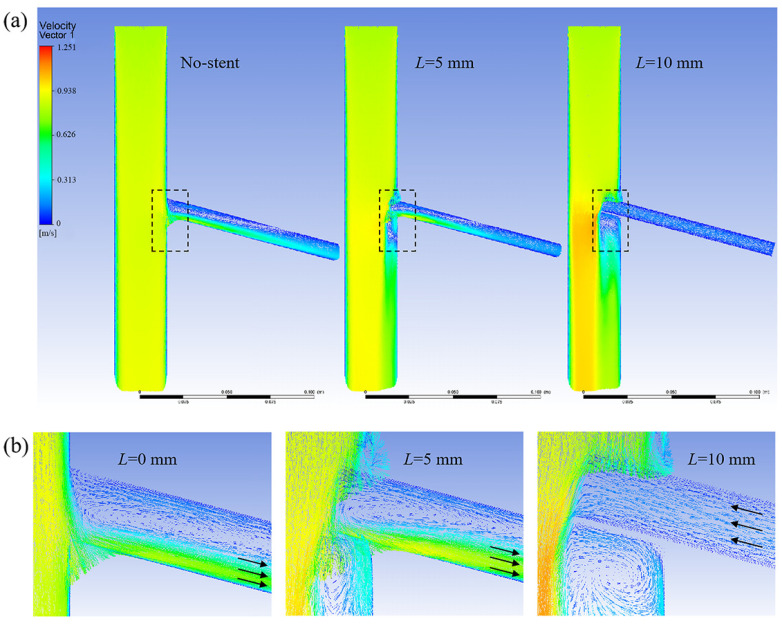
(**a**) Model velocity distribution cloud chart at a tilt angle of 105°; (**b**) Enlarged views of the branching area within the dashed box.

Figure 6 shows a model velocity distribution cloud chart for different branch entry depths at a tilt angle of 105°. At a branching depth of 5 mm, the branch wall impeded the blood flow, and vortex flow was generated below the stent. However, at this time, the branch stent was tilted at a large angle, and the direction of the stent was the same as the direction of the aortic blood flow, so the blood flow in the branch artery still presented an outflow state.

**Figure 7:**
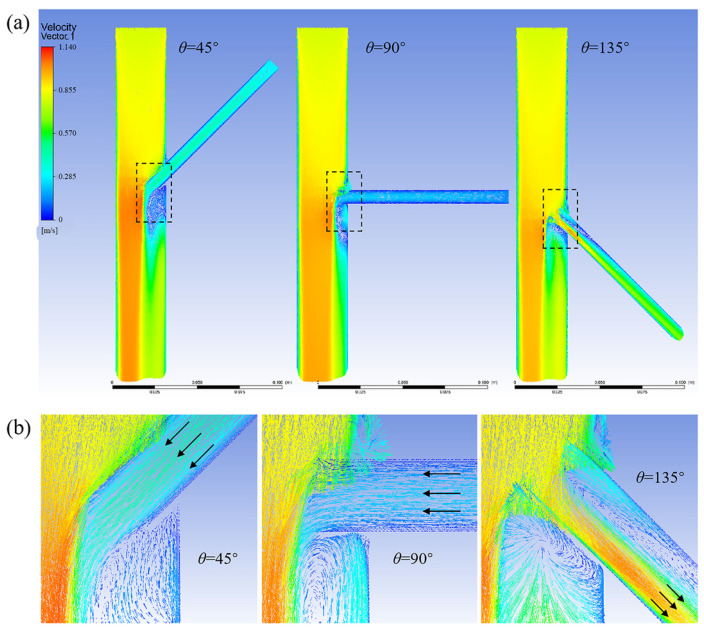
(**a**) Model velocity distribution cloud at a 10 mm entry depth; (**b**) Enlarged views of the branching area within the dashed box, arrows represent the direction of blood flow.

Figure 7 shows a cloud chart of the velocity distribution of the model at different tilt angles for an entry depth of 10 mm. When the tilt angle is 45°, the branch stent is oriented in the opposite direction of aortic blood flow, and the obstruction of blood flow by the branch wall is most obvious, resulting in blood flow reflux from the branch stent. As the tilt angle increases, the direction of the branch stent gradually conforms to the direction of aortic blood flow, and the reflux from the branch decreases until it turns into an outflow state. Therefore, increasing the tilt angle can effectively change the vortex condition and, thus, the blood flow status.

#### 3.1.3. Relationship Between Branch Flow and Branch Stent Diameter

**Figure 8:**
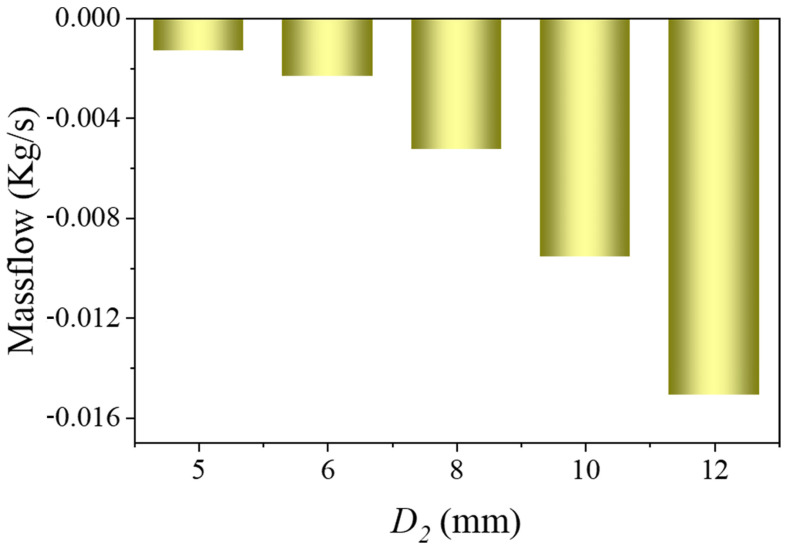
Relationship between branch flow and branch stent diameter (D1 = 30 mm, θ = 90°, L = 15 mm, Vinlet = 0.4 m/s).

Figure 8 shows the relationship between branch flow and branch diameter. When the aortic diameter and branch entry depth were fixed, the reflux volume increased with the diameter of the branch stent.

**Figure 9:**
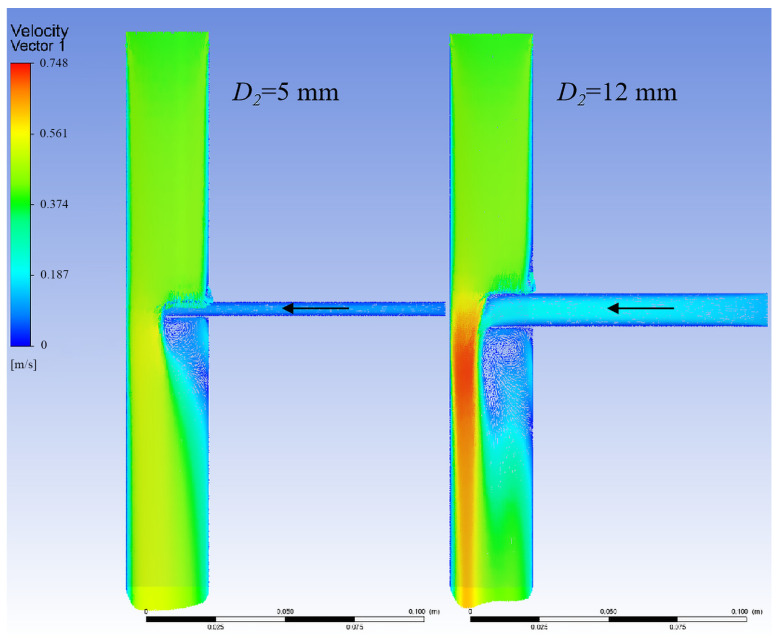
Model velocity distribution cloud for an aortic diameter of 30 mm, arrows represent the direction of blood flow.

Figure 9 displays the model velocity distribution cloud chart for different branch stent diameters with an aortic diameter of 30 mm. As the diameter of the branch stent increased, the vortex region became larger, and the branch return flow rate increased.

#### 3.1.4. Optimal Design of the Branch Stent

**Figure 10:**
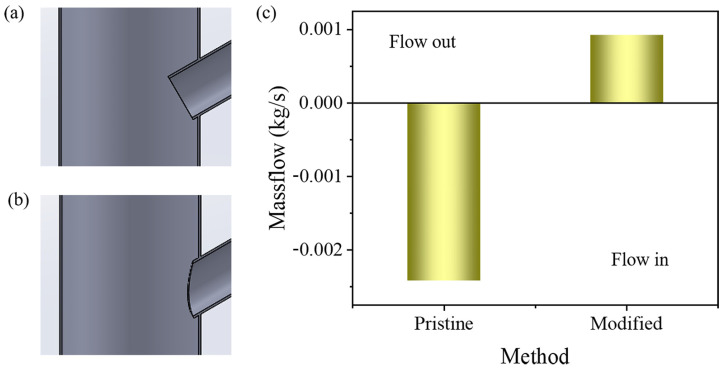
Comparison of branch flow rates in the initial and modified stents. (**a**) Initial stent. (**b**) Modified stent. (**c**) Comparison of branch flows under the two conditions.

The above analysis reveals that the branch canal wall created a significant obstruction to blood flow. To improve blood flow and reduce the stent’s impact on blood flow, some modifications to the initial model were made using partial resection (see Figure 10a,b). As depicted in Figure 10c, blood flow exhibited a reflux state with the original stent and an outflow state with the optimized stent. This indicates that blood flow movement can be effectively changed from reflux to outflow by means of partial excision.

### 3.2. Case Model Simulation Results

Table 1 lists demographic data, postoperative renal artery status, and flow-affecting parameters in six patients after F/B-EVAR. Patients A, B, and C had patent renal arteries. Patient D developed bilateral renal artery occlusion, while Patients E and F had postoperative left renal artery occlusion. All six patients had similar follow-up blood pressure values and blood viscosity impact indicators within normal ranges. Therefore, the same calculation conditions were used for flow simulation.

**Figure 11:**
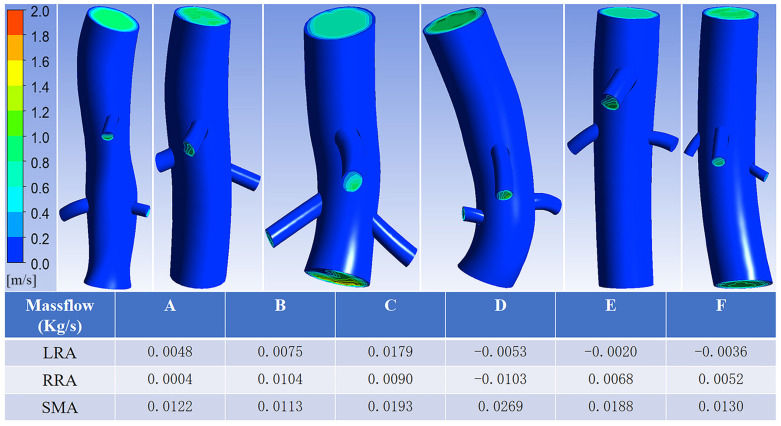
Calculated results of the stent model for six patients (LRA: left renal artery; RRA: right renal artery; SMA: superior mesenteric artery).

Since the patients with postoperative branch artery occlusion were all renal arteries, and the middle renal artery blood flow velocity and pressure were selected as the reference for setting the boundary conditions, the discussion focuses on the relationship between the branch entry conditions and the outflow of bilateral renal artery stents in six patients. The postoperative renal artery patency patients and the postoperative renal artery occlusion patients were divided into patency and occlusion groups, respectively. Table 2 and Table 3 show the stenting conditions of the aortic and bilateral renal artery stents in the patency and occlusion groups, respectively, and Figure 11 illustrates the results of the in-stent flow calculations in the six patients.

According to the simulation results, both renal arteries of Patient D were in a reflux state, and the left renal arteries of Patients E and F were also in a reflux state, while the rest of the renal arteries were in an outflow state. This aligns with the actual post-surgery situation, where the right and left renal arteries of Patient D were occluded, and the left renal arteries of Patients E and F were also occluded, whereas the rest of the renal arteries remained patent.

A comparison of the postoperative occluded and patent renal artery stenting conditions revealed that the stenting angle was smaller in all renal arteries with postoperative occlusion (77.5–91.9°) and larger in all postoperative patent renal arteries (103.5–139.8°). However, the differences in entry depth and stent diameter in the occluded renal arteries compared with the patent renal arteries were not significant.

Differential analysis of the tilt angle, entry depth, and branch stent diameter was performed in the six patients (Table 4). The median outflow of the 12 renal arteries was 0.005 Kg/s, and the renal arteries were divided into two groups based on the median. The renal arteries with outflow less than this value were set up as the low-flow group, those with outflow greater than this value were set up as the high-flow group, and the number of renal arteries in each group was assigned to be six. The effect size of the tilt angle between the two groups was 1.32, with *p* = 0.004; the effect size of the entry depth between the two groups was 0.18, with *p* = 0.522; and the effect size of the renal artery stent diameter between the two groups was 0.05, with *p* = 0.873. This suggests that the tilt angle is a key factor influencing renal artery flow.

**Figure 12:**
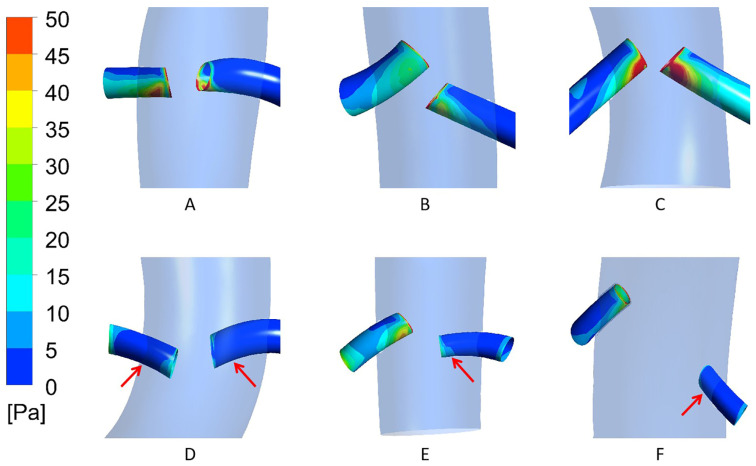
WSS distribution in patent and occluded renal arteries of (**A**–**F**) six patients (red arrows indicate occluded renal artery).

Based on the analysis of renal artery blood flow, we further evaluated the distribution of WSS in the renal arteries of the six patients. As shown in Figure 12, compared with the patent renal arteries, the occluded renal arteries exhibited lower WSS values. This finding is consistent with the previous research results on renal artery blood flow.

In light of this study, we recommend the following strategies for F/B-EVAR of abdominal aortic aneurysms: During preoperative planning, prioritize the release path of renal artery stents with a tilt angle greater than 100°. During the operation, if the tilt angle is limited, compensation can be achieved by dilating the opening of the renal artery stent with an intravascular balloon or reducing the insertion depth. After the operation, intensify the imaging follow-up of patients whose renal artery stent tilt angle is less than 90° to identify the occlusion risk at an early stage.

## 4. Discussion

Traditional open surgery has been the gold standard in the treatment of thoraco-abdominal aortic aneurysm (TAAA) since Etheredge et al. reported the first open surgery for TAAA in 1955 [3], but patients undergoing such surgery face a high incidence of serious complications, including renal failure and paraplegia, as well as a high mortality rate [4,5].

Various solutions to this challenge, such as hybrid surgery, multiple chimney technology and F/B EVAR, have emerged in recent years. Hybrid surgery still requires an open procedure to temporarily block blood flow to the internal arteries, making it unsuitable for elderly patients, and does not offer any significant advantages over conventional surgery [6,7]. The multiple chimney technique (including the octopus technique) is a fully minimally invasive approach with a relatively short procedure time and simple technique [8]. However, this technique uses a long, covered stent as a branch of the visceral artery, leading to gaps and compression between the stents. This non-physiological approach results in a high incidence of endoleaks and occlusion of branch artery stents during long-term follow-up [9,10,11].

Based on the patient’s anatomy, F/B EVAR is the standard physiological method for total endovascular aneurysm repair of the TAAA. Its primary advantages include the absence of aortic blockage and a short duration of visceral ischemia. According to the available literature, the use of F/B EVAR is associated with a significantly lower mortality and complication rate compared to traditional open and hybrid surgery for TAAA, making it suitable for elderly and frail patients with severe comorbidities [12,13]. In order to further optimize the F/B EVAR technique and to reduce the incidence of the serious complication of postoperative renal dynamic occlusion [14], we conducted this study.

The calculations of the theoretical model show that larger tilt angles, smaller branch entry depths, and larger branch stent diameters, all of which favor normal blood flow in branch arteries. On this basis, by improving the form of the proximal end of the branch stent and preparing it in a beveled configuration, so that the stent entrance is parallel to the aortic wall when the stent is placed, which can greatly optimize the blood flow in the branch artery. The calculation results of the actual case model, on the other hand, showed that there was a significant difference in the tilt angle of the renal artery stent between the high- and low-flow groups (*p* = 0.004), whereas there was no significant difference in entry depth (*p* = 0.522) and branch stent diameter (*p* = 0.873). This indicates that the tilt angle of the branch stent is a significant influencing factor of branch arterial flow under the current equipment and technical conditions.

In addition, the calculation results of WSS show that the WSS value of the occluded renal arteries was significantly lower than that of the patent renal arteries. Existing studies have shown that regions with low WSS tend to have lower blood flow velocities [15]. Moreover, other research findings indicate that thrombus formation is more likely to occur in low-WSS regions [16], which may lead to renal artery occlusion. These are consistent with the results of our study, suggesting that low WSS may be a predictor of renal artery occlusion.

Compared with existing studies, this research verifies the impact of renal artery stent implantation conditions on renal artery blood flow through the combination of theoretical models and patient-specific models. Previous similar studies only used idealized theoretical models [17,18]. Our study not only reproduced their results but also enhanced the reliability of the findings through patient-specific models. On this basis, we have proposed corresponding clinical operation recommendations for preoperative planning, intraoperative operations, and postoperative monitoring, providing guidance for doctors.

Considering that multiple factors, such as the tilt angle, entry depth, and stent diameter, may simultaneously affect renal artery blood flow, in theory, multivariate regression can be used to deeply analyze the combined effects of these factors. However, the sample size of this study was relatively small (only six cases). Using multivariate regression may lead to overfitting and fail to accurately reflect the true variable relationships. In future research, we will recruit more samples and use multivariate regression to explore the comprehensive effects of various factors, revealing the mechanism of renal artery blood flow changes more comprehensively.

Compared with transient simulations, the steady-state simulation conditions used in this study lack a realistic reflection of physiological conditions and may underestimate the dynamic changes of hemodynamic parameters [15,19]. However, the focus of this study was to evaluate the impact of stent geometric parameters (such as tilt angle, entry depth, etc.) on renal artery blood flow, rather than time-dependent dynamic behaviors. In previous studies on stent geometric parameters, there were precedents for using steady-state simulation conditions, and these studies could meet the research objectives, demonstrating the applicability of steady-state simulations within a specific research scope [20,21].

This study adopted the rigid-wall assumption, which has been widely used in researching the hemodynamic changes after endovascular aneurysm repair (EVAR) of the abdominal aorta [22,23]. Existing studies have shown that when researching the hemodynamic parameters of the abdominal aorta, the calculation results of the rigid-wall model and the fluid–structure interaction (FSI) model have small differences [22,24]. Therefore, under such circumstances, using a rigid wall can simplify the model, save calculation time, and still provide reasonable approximations [25]. However, the rigid-wall model ignores the interaction between blood flow and vascular wall deformation, and its prediction accuracy has certain limitations. It is more suitable for preliminary evaluation and qualitative analysis.

To further improve the accuracy and comprehensiveness of the research, in the future, we will introduce transient simulations and fluid–structure interaction (FSI) models for more in-depth analysis. By combining these two methods, we expect to more comprehensively reveal the impact mechanism of stent geometric parameters on renal artery blood flow and provide a more reliable theoretical basis for clinical practice.

## 5. Conclusions

The use of F/B EVAR for TAAA is a more physiologically normal approach with lower postoperative mortality and complication rates than traditional open surgery and hybrid surgery. However, during F/B EVAR, there is often a partial branch artery stent extending into the aortic stent. This change in structure and shape can affect the state of blood flow within the stent, which, in turn, may lead to restenosis or occlusion of the branch arteries. Our study constructed a 3D model of an artificial stent placed in the aorta, reflecting the actual clinical procedure. Using computational fluid dynamics, we visually depicted the changes in blood flow under different conditions. By analyzing the effects of stent entry depth, tilt angle, inlet blood velocity, aortic diameter, and branch stent diameter on blood flow in the branch stent, we can draw concise and regular conclusions. For instance, the branch stent is conducive to improving branch artery patency when it conforms to the direction of aortic blood flow. These findings will help guide the placement of stents in clinical procedures.

## Figures and Tables

**Table 1:** Demographic data, postoperative renal artery status, and blood flow-affecting parameters in six patients.

Patient	Patient A	Patient B	Patient C	Patient D	Patient E	Patient F
Renal artery	Patent	Patent	Patent	Bilateral occlusion	Left occlusion	Left occlusion
Blood pressure	70/138 mmHg	72/130 mmHg	74/133 mmHg	63/138 mmHg	71/141 mmHg	70/135 mmHg
Erythrocyte value	4.66 × 10^12^/L	6.24 × 10^12^/L	4.34 × 10^12^/L	4.56 × 10^12^/L	4.44 × 10^12^/L	4.51 × 10^12^/L
Leukocyte value	5.6 × 10^9^/L	5.4 × 10^9^/L	5.2 × 10^9^/L	5.1 × 10^9^/L	7.5 × 10^9^/L	4.9 × 10^9^/L
Platelet value	103 × 10^9^/L	140 × 10^9^/L	237 × 10^9^/L	156 × 10^9^/L	171 × 10^9^/L	224 × 10^9^/L
Triglyceride value	1.66 mmol/L	0.98 mmol/L	0.68 mmol/L	0.83 mmol/L	1.27 mmol/L	0.88 mmol/L
Total cholesterol	3.82 mmol/L	4.03 mmol/L	4.40 mmol/L	3.77 mmol/L	3.64 mmol/L	4.62 mmol/L

**Table 2:** Bilateral renal artery stent branching conditions in patients in the patency group (LRA: left renal artery; RRA: right renal artery).

Patient Conditions	Patient A		Patient B		Patient C	
	LRA	RRA	LRA	RRA	LRA	RRA
Tilt Angle	103.5°	105.6°	131.8°	126.7°	139.8°	106.7°
Entry Depth	5.04 mm	10.06 mm	8.59 mm	7.88 mm	9.55 mm	6.16 mm
Branch Diameter	5.21 mm	5.10 mm	4.95 mm	5.72 mm	5.50 mm	5.50 mm
Aortic Diameter	23.21 mm		19.14 mm		21.77 mm	

**Table 3:** Bilateral renal artery stent branching conditions in patients in the occlusion group.

Patient Conditions	Patient D		Patient E		Patient F	
	LRA	RRA	LRA	RRA	LRA	RRA
Tilt Angle	81.1°	77.5°	91.9°	118.8°	80.5°	127.1°
Entry Depth	6.34 mm	10.13 mm	6.75 mm	4.59 mm	5.67 mm	4.80 mm
Branch Diameter	5.80 mm	5.47 mm	3.88 mm	3.67 mm	3.92 mm	4.20 mm
Aortic Diameter	34.93 mm		18.06 mm		21.88 mm	

**Table 4:** Differential analysis of high and low mass flow groups.

	High Mass Flow (n = 6)	Low Mass Flow (n = 6)	Cohen’s d	95%CI	*p*
Tilt Angle (°)	126.9 (115.8, 133.8)	86.5 (79.8, 104.0)	1.32	0.89–1.75	0.004
Entry Depth (mm)	7.02 (4.75, 8.83)	6.55 (5.51, 10.03)	0.18	−0.35–0.71	0.522
Branch Diameter (mm)	5.23 (4.07, 5.56)	5.16 (3.91, 5.56)	0.05	−0.48–0.58	0.873

## Data Availability

The data underlying this article will be shared upon reasonable request to the corresponding authors.
